# Effects of traditional Chinese exercise on the treatment of COVID-19: A protocol for systematic review

**DOI:** 10.1097/MD.0000000000028948

**Published:** 2022-02-25

**Authors:** Li Chen, Yu Ji, QiPeng Wang, MingFen Fu

**Affiliations:** a Chengdu Sport University, Chengdu, Sichuan Province, China; b Sichuan Normal University, Chengdu, Sichuan Province, China; c Affiliated Sport Hospital of Chengdu Sport University, Sichuan Province, China.

**Keywords:** corona virus disease 2019, systematic review, traditional Chinese exercise

## Abstract

**Introduction:**

Since the end of December 2019, corona virus disease 2019 (COVID-19) has caused a huge impact in many countries and has attracted great attention from countries around the world. In fact, many studies have shown that during the fight against the COVID-19 epidemic. Chinese traditional exercise plays an active role in promoting human health. The main purpose of this study is to provide a reliable method and credible evidence to improve the prognosis of patients with COVID-19 through traditional Chinese exercise.

**Methods:**

This protocol is guided by the preferred reporting items for systematic reviews. By searching the following electronic databases: PubMed, EMBASE, MEDLINE, Cochrane Library, Web of Science, China National Knowledge Infrastructure Database, China Biomedical Literature Database, China Science, and Wan-Fang Database. The whole process includes selecting high-quality literature, extracting and analyzing, and assessing the risk of bias in order to summarize the therapeutic effect of traditional Chinese exercise on COVID-19 patients.

**Result:**

Research shows that prevention and treatment through traditional Chinese exercise can provide strong evidence against COVID-19.

**Conclusion:**

To provide a way to help prevent and treat COVID-19 through traditional Chinese exercise.

## 1 Introduction

In December 2019, the corona virus disease 2019 epidemic broke out in Wuhan.^[1]^ The World Health Organization (WHO) named this virus-infected pneumonia “COVID-19.” The WHO has declared the ongoing corona virus disease 2019 (COVID-19) epidemic as a global public health emergency and a highly infectious disease.^[2,3]^ As of December 3, 2021, the number of confirmed cases of COVID-19 reported to the WHO worldwide is 263,563,622, including 5,232,562 deaths.^[4]^ According to recent evidence, it has been observed that COVID-19 is an infection caused by the severe acute respiratory syndrome corona virus 2 (SARS-CoV-2). SARS-CoV-2 is one of the coronaviruses, which can spread from person to person.^[5]^ SARS-CoV-2 is widely regarded as a serious traumatic event, which poses a threat not only because of physical problems, but also because of the psychological distress of infected patients.^[6]^ Traditional Chinese exercise (TCE), with a long history, it is a combination of movement and static, rigid, and flexible movement mode, represented by Tai Chi, Baduanjin, Wu Qin Xi, Yi Jin Jing, and so on. Tai Chi is one of the Chinese martial art. It is composed of light to moderate aerobic exercise, combining physical and mental training.^[7]^ At present, Tai Chi training can be used as a pulmonary rehabilitation program and has a positive effect on patients with chronic obstructive pulmonary disease.^[8,9]^ For patients, rehabilitation exercise and abdominal breathing training exercise therapy can effectively increase the patient's exhalation capacity and improve lung function.^[10]^ Therefore, we will study the effect of traditional Chinese exercise on the prognosis and treatment effect of COVID-19 patients in a systematic review and analysis.

## 2 Methods

### 2.1 Registration

This study protocol systematic review has been registered in INPLASY. The registration number is INPLASY202210089.

### 2.2 Inclusion criteria

#### 2.2.1 Study designs

We will include research related to traditional Chinese exercise therapy for COVID-19 patients. In order to obtain a more objective and true evaluation of the research, all the referenced documents must meet the following 4 conditions at the same time: published documents with complete documents data; the research subject is confirmed to have COVID-19; the intervention group received traditional Chinese exercise therapy intervention for at least a period of treatment; including randomized controlled trials, controlled (non-randomized) clinical trials or cluster trials, post-control studies, prospective and retrospective comparative cohort studies, and case-control or nested case-control studies. Only when there are at least 2 intervention sites and 2 control sites will it be included in the controlled before-after study. We will exclude cross-sectional studies, case series, and case reports.

#### 2.2.2 Participants

Regardless of gender, age, race, or education and economic status, COVID-19 patients who have been clearly diagnosed and are in recovery will be included. Postoperative infections, psychiatric patients, severe pneumonia, or patients unable to exercise due to other reasons will not be included.

#### 2.2.3 Interventions

The experimental group will be divided into 4 groups, treated with TCE, including Wuqinxi, Baduanjin, Yijinjing, Tai Chi, and so on. The control group is treated with non-TCE or pharmacotherapies. Pharmacotherapies include drugs recommended in international or domestic authorized clinical guidelines. Studies which combine TCE with pharmacotherapy are required to use the same pharmacotherapy in both the experimental and the control groups.

#### 2.2.4 Comparators

Comparisons include rest, psychosocial therapy, and drug therapy. In addition, the study will also include studies that compare traditional Chinese exercise therapy with another method of treatment alone.

#### 2.2.5 Outcomes

Primary outcomes: The disappearance of the main symptoms (including fever, cough, a nucleic acid test, body temperature recovery time) and physical function indicators (blood pressure, heart rate, body composition, muscle strength, joint mobility, activities of daily living) disappeared.

Secondary outcome: The disappearance of the accompanying symptoms (such as myalgia, chest tightness, runny nose, headache, nausea, vomiting, and diarrhea); COVID-19 nucleic acid test results for 2 consecutive times (not the same day) negative result rate, CT image improvement, common type to incidence rate, clinical cure rate, and mortality rate of severe illness.

### 2.3 Data sources

The following electronic databases will be searched to identify relevant research: PubMed, EMBASE, MEDLINE, Cochrane Library, Web of Science, China National Knowledge Infrastructure Database (CNKI), China Biomedical Literature Database (CBM), China Science and Wan-Fang Database. Search dates: from inception dates to December 2021.

### 2.4 Data collection and analysis

#### 2.4.1 Search strategy

The search terms on PubMed are as follows: “Traditional Chinese exercise (e.g., Taijiquan, Baduanjin, Wuqinxi, Yijinjing)”;“COVID-19” OR “Corona Virus Disease 2019” OR “Novel Corona Virus” OR “2019- nCoV”; “convalescence” OR “Rehabilitation” OR “recovery”; “randomized controlled trial” OR “randomized” OR “randomly” OR “clinicaltrial.” A combination of medical subject headings (MeSH) and free text words will be used. The same search terms are used in other electronic databases. These search terms are shown in Table 1.

**Table 1:** Search strategy for the PubMed database.

Number	Search items
1	Traditional Chinese exercise
2	“Tai Chi” OR “Baduanjin” OR “Wu Qin Xi” OR “ Yi Jin Jing”
	1 or 2
3	COVID-19
4	Corona Virus Disease 2019
5	Novel Corona Virus
6	2019- nCoV
7	4 or 5–7
8	Convalescence
9	Rehabilitation
10	Recovery
11	9 or 10–11
12	randomized controlled trial
13	randomized
14	randomly
15	clinicaltrial
16	13 or 14–16
17	3 and 8 and 12 and 17

#### 2.4.2 Study selection

**Figure 1:**
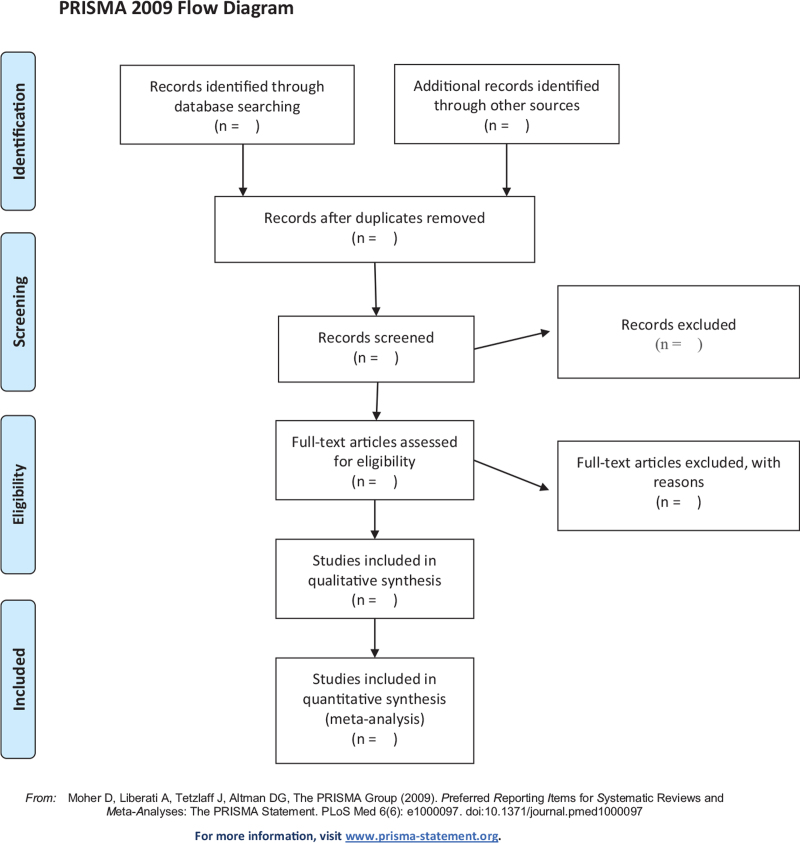
Flow chart of the study. Adapted from Preferred Reporting Items for Systematic Reviews and Meta-Analyses Protocols (PRISMA-P).

Documents will be retrieved according to the retrieval strategy. Before retrieval, all reviewers will discuss and determine the selection criteria, and then exclude some duplicate documents or documents with incomplete information. Finally, the document records will be imported into EndNoteX9 (Thomson Corporation, Stanford, Connecticut) software for management. The above steps were performed independently by 2 researchers. Any disagreements will be resolved through discussions with the third researcher. Researchers will record all documents that do not meet the inclusion criteria and provide reasons for excluding them. We chose the preferred reporting items for systematic reviews and meta-analyses (PRISMA) flow chart to show the entire process of selecting documents for the study (Fig. 1).

#### 2.4.3 Data extraction

Extract data based on selected studies and record them in an EXCEL file, which includes the following items: name of the main author, name of the publication journal, publication year, study sample size, intervention methods, intervention results, bias risk assessment, and results. The results will be carried out independently by 2 researchers, and any differences will be resolved through discussions with a third researcher. If the required information is not reported, we will contact the corresponding author by phone or email to obtain more information to deal with the missing data.

#### 2.4.4 Risk of bias assessment

The 2 researchers will use Cochrane risk of bias tool to evaluate the methodological quality of the included studies. We will consider the following items: random sequence generation (selection bias); allocation hiding (selection bias); blinding of subjects and staff (performance bias); outcome evaluation blinding (detection bias); incomplete outcome data (Attrition bias); selective reporting (reporting bias); other sources of bias (other biases). The risk of bias in all aspects of the research is evaluated, divided into 3 levels: low risk, high risk, and unclear risk.

#### 2.4.5 Measures of treatment effect

In this agreement, we will use the 95% confidence interval hazard ratio to strictly analyze the binary data. For continuous data, the mean difference or standard mean difference is used to measure the efficacy of 95% confidence interval.

#### 2.4.6 Data synthesis

Each result will be calculated and combined using RevMan 5.3. The specific implementation is based on the current version of the Cochrane Intervention System Evaluation Manual. If the heterogeneity test is not significant, the Mantel–Haenszel method is selected as the fixed effects model, and if statistical heterogeneity is observed (*I*^2^* *≥* *50% or *P* < .1), the random effects model is used. If the heterogeneity is substantial, we will make a narrative qualitative summary. We will clearly describe which studies are included and how to synthesize them as described. We will be transparent about the indicators used.

#### 2.4.7 Subgroup analysis

We will conduct a subgroup analysis based on different patient characteristics, intervention methods (traditional Chinese exercise), treatment duration, and outcome indicators.

#### 2.4.8 Sensitivity analysis

Sensitivity analysis is to analyze the quality of research, intervention methods, types, etc, and eliminate the researches with quality defects one by one, so that we can determine the source of heterogeneity.

#### 2.4.9 Grading the quality of evidence

This study will use the evidence quality grading to evaluate the results of the study. The results will be divided into 4 evaluation levels: high, medium, low, and very low.

### 2.5 Ethics and dissemination

The content of this article does not involve ethical recognition or ethical review. The research results will be submitted to relevant journals or conferences for publication and information sharing.

## 3 Discussion

This article will evaluate the therapeutic effects of traditional Chinese exercise on COVID-19, including: inclusion criteria, data sources, data collection, analysis, etc. This study can provide some method guidance for other scholars to study the effects of traditional Chinese exercise on the prevention and treatment of COVID-19. However, due to some limitations, this study also has shortcomings.

## 4 Limitations

Due to the extremely variant strain of COVID-19, a virus for the whole world, we should pay attention to non-English language analysis in order to ensure the accuracy of the conclusion, so that other scholars can further study COVID-19.

## Author contributions

**Conceptualization:** Li Chen.

**Data curation:** Yu Ji, Qi Peng Wang, MingFen Fu.

**Formal analysis:** Li Chen, Yu Ji, Qi Peng Wang.

**Funding acquisition:** Li Chen.

**Methodology:** Qi Peng Wang.

**Software:** Yu Ji, Qi Peng Wang, MingFen Fu.

**Writing – original draft:** Li Chen.

**Writing – review & editing:** Yu Ji, Qi Peng Wang.
